# *KMT2A‐D* pathogenicity, prevalence, and variation according to a population database

**DOI:** 10.1002/cam4.5443

**Published:** 2022-12-08

**Authors:** Jenna K. Larson, DeVon N. Hunter‐Schlichting, Erin L. Crowgey, Lauren J. Mills, Todd E. Druley, Erin L. Marcotte

**Affiliations:** ^1^ Deparatment of Genetic Counseling University of Minnesota Minneapolis Minnesota USA; ^2^ Masonic Cancer Center University of Minnesota Minneapolis Minnesota USA; ^3^ Division of Pediatric Epidemiology and Clinical Research, Department of Pediatrics University of Minnesota Minneapolis Minnesota USA; ^4^ Nemours Children's Health Wilmington Delaware USA; ^5^ Mission Bio South San Francisco California USA; ^6^ Brain Tumor Program University of Minnesota Minneapolis Minnesota USA

**Keywords:** epidemiology, genetic variants, human genetics, leukemia

## Abstract

**Introduction:**

The *KMT2* family of genes is essential epigenetic regulators promoting gene expression. The gene family contains three subgroups, each with two paralogues: KMT2A and KMT2B; KMT2C and KMT2D; KMT2F and KMT2G. *KMT2A‐D* are among the most frequent somatically altered genes in several different cancer types. Somatic *KMT2A* rearrangements are well‐characterized in infant leukemia (IL), and growing evidence supports the role of additional family members (*KMT2B*, *KMT2C*, and *KMT2D*) in leukemogenesis. Enrichment of rare heterozygous frameshift variants in KMT2A and C has been reported in acute myeloid leukemia (AML), IL, and solid tumors. Currently, the non‐synonymous variation, prevalence, and penetrance of these four genes are unknown.

**Methods:**

This study determined the prevalence of pathogenic/likely pathogenic (P/LP) germline *KMT2A‐D* variants in a cancer‐free adult population from the Genome Aggregation Database (gnomAD). Two methods of variant interpretation were utilized: a manual genomic variant interpretation and an automated ACMG pipeline.

**Results:**

The ACMG pipeline identified considerably fewer P/LP variants (*n* = 89) compared to the manual method (*n* = 660) in all 4 genes. Consequently, the total P/LP prevalence and allele frequency (AF) were higher in the manual method (1:112, AF = 4.46E‐03) than in ACMG (1:832, AF = 6.01E‐04). Multiple ancestry‐exclusive P/LP variants were identified along with an increased frequency in males compared to females. Many of these variants identified in this population database are also associated with severe juvenile conditions.

**Conclusion:**

These data demonstrate that putatively functional germline variation in these developmentally important genes is more common than previously appreciated and identification in cancer‐free adults may indicate incomplete penetrance for many of these variants. Future research should examine a genetic predisposing role in IL and other pediatric cancers.

## Graphical abstract

This analysis highlights the pathogenic spectrum of heterozygous KMT2A, KMT2B, KMT2C, and KMT2D mutation found in the gnomAd database, utilizing the non‐cancer adult cohort. Pathogenic mutations associated with severe pediatric cancer and genetic syndromes were identified in an ostensibly healthy adult. This analysis provides insight into the prevalence and penetrance of these pathogenic mutations in the general population.

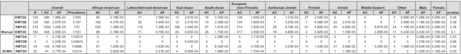

## 1 INTRODUCTION

The histone–lysine *N*‐methyltransferase 2 (*KMT2*) family of genes, previously known as mixed lineage leukemia (MLL), encode for H3K4 methyltransferases that play an essential role in epigenetic regulation of gene expression via altering chromatin structure to promote DNA accessibility.^1^, ^2^ The six gene members in this family *KMT2A* [MIM: 159555], *KMT2B* [MIM: 606834], *KMT2C* [MIM: 606833], *KMT2D* [MIM: 602113], *KMT2F* (*SETD1A* [MIM: 611052]), and *KMT2G* (*SETD1B* [MIM: 611055]) are highly conserved among eukaryotes given their importance in cellular function and mammalian gene expression during early development.^1^, ^2^ These four genes encode for large proteins part of complexes crucial for transcription, known as COMPASS (COMplex of Proteins ASsociated with SET1).^3^ Beyond the well‐characterized somatic dysregulation resulting from *KMT2A*‐rearranged pediatric leukemia, recent studies have shown *KMT2A‐D* variants are indeed among the most frequent genomic alterations in a variety of human malignant neoplasms with associations to common blood and solid tumor cancers.^2^, ^3^ Genotype–phenotype correlations have been established between *KMT2A* (11q23.3), *KMT2B* (19q13.12), *KMT2C* (7q36.1), and *KMT2D* (12q13.12) somatic variants and different cancer types.^2^, ^3^, ^4^

*KMT2A*‐related cancers are strongly associated with large somatic chromosomal rearrangements with over 100 translocation partners creating fusion proteins linked to disease phenotypes of particularly aggressive adult and infantile leukemias.^2^, ^5^ Emerging data on *KMT2B* has found potential associations between somatic short pathogenic variants and hepatocellular carcinoma as well as translocations with undifferentiated spindle cell sarcomas.^6^, ^7^ However, *KMT2B* has yet to be as confidently identified as a driver of oncogenesis.^8^ Advancements in cancer exome sequencing studies have supported somatic *KMT2C* haploinsufficiency in acute myeloid leukemia, pancreatic ductal carcinoma, bile duct carcinoma, cutaneous squamous cell carcinoma, gastric adenocarcinoma, and hepatocellular carcinoma.^9^ Similar studies have shown somatic *KMT2D* variants to be associated with non‐Hodgkin lymphoma, renal carcinoma, squamous cell carcinomas, mantle cell lymphoma, and pediatric tumors more generally.^9^ All these associations are additionally complicated by potential polygenic combinatorial effects causing a statistically significant co‐occurrence rate, suggesting variants in several *KMT2* genes may be required for the development of various cancer types.^3^ Beyond the malignant neoplasm associations, there are additional correlations with autosomal dominant congenital disorders resulting from *de novo* heterozygous germline variants^3^: *KMT2A* with Wiedemann‐Steiner syndrome (WDSTS [MIM: 605130]),^10^ *KMT2B* with pediatric dystonia,^11^ *KMT2C* with Kleefstra syndrome (KLEFS1 [MIM: 610253]),^12^, ^13^ and *KMT2D* with Kabuki syndrome (KABUK1 [MIM: 147920]).^11^, ^14^

Of particular interest is the relation of the *KMT2* genes to pediatric cancers, such as IL. IL describes children diagnosed with either acute lymphocytic leukemia (ALL) or acute myeloid leukemia (AML) up to one year of age.^15^, ^16^ This sporadic, rare cancer (approximately 150 cases diagnosed in the United States annually) has a poor prognosis with a 5‐year event‐free survival rate of approximately 50%.^16^, ^17^ Additionally, those who survive initial cancer frequently experience lifelong late effects such as developmental and cognitive deficits.^18^, ^19^ Such lack of progress likely results from an inadequate understanding of IL given the unique natural history and epidemiology of this disease compared to the other pediatric leukemia types, suggestive of novel biology.^20^ Further, IL has rapid onset *in utero* and is subsequently understood to result from improper hematopoietic development.^21^

One of the defining features of IL is the disproportionate presence of *KMT2A* chromosomal rearrangements.^22^, ^23^ Such translocations are seen in ~34%–50% of infant AML cases^24^ and approximately 80% of ALL cases,^25^ however, new evidence suggests additional factors are needed to fully explain leukemogenesis.^3^, ^26^ With one of the lowest known rates of somatic variants beyond *KMT2A* rearrangements compared to any other cancer type, it is now hypothesized that inherited germline variation (notably through an excess of rare, non‐synonymous variants) may lead to the development of IL.^26^ One study, building off an observed enrichment of germline missense variants in *KMT2C*, found complete loss of this epigenetic regulator hinders mesoderm to hematopoietic specification in human pluripotent cells (hPSCs) *in vitro*.^26^, ^27^ Thus, germline variants and alterations in other *KMT2* genes, independent of *KMT2A* translocations, must be further explored to gain a more comprehensive look at IL predisposition.

The recent availability of large‐scale genomic data from publicly available population datasets, such as the Genome Aggregation Database (gnomAD), allows an estimation of pathogenic germline variation within specific genes to be obtained.^28^ Particularly, gnomAD provides insight on individuals screened for no history of childhood disease (i.e., cancer or cardiology condition, etc.) among participants or in any of their first‐degree relatives, encompassing a wide range of ages (18–85 years), ancestry, and sex assigned at birth information. Understanding the non‐synonymous variation, P/LP prevalence, and frequencies of germline heterozygous *KMT2* variants in a cancer‐free adult population could supplement the broader understanding of how different genetic variants contribute to the phenotype of certain conditions. Germline heterozygous variants in these four genes are associated with severe congenital and pediatric conditions.^10^, ^26^, ^29^, ^30^ Hence, this analysis may provide insight into the penetrance of these variants seen in cancer‐free adults. To our knowledge, this is the first description of the variation of pathogenic heterozygous germline *KMT2A‐D* variants in a large population database.

## 2 MATERIALS AND METHODS

### 2.1 Large, publicly available datasets in gnomAD

Version 3 (v3.1.2) of gnomAD was used to examine *KMT2A‐D* non‐synonymous sequence germline variation in individuals within non‐cancer datasets as there is an overall cancer predisposition in pathogenic *KMT2* variants. Due to the larger sample size of African American individuals and the inclusion of Amish and Middle Eastern populations, version 3 was chosen over version 2. These populations span both whole genomes and exomes from unrelated individuals mapped to the GRCh38 build of the human reference genome. Participant genomes and exomes were sequenced as part of both population‐wide and disease‐specific genetic studies and had no personal history of common disease, or known cardiac, cancer, or neurologic diagnosis. After gnmoAD's internal sequencing QC filtering^28^ was applied, each gene had between 74,019 and 74,032 individuals included in this analysis (Table S1). The use of non‐cancer datasets confirmed the variants in this analysis were not from individuals ascertained to have cancer in cancer studies allowing a closer look at the baseline frequency of variation in a cancer‐free general population.

All exonic, splice‐site region, and intronic variants from the non‐canonical *KMT2A* (NM_001197104.2), *KMT2B* (NM_014727.3), *KMT2C* (NM_170606.3), and *KMT2D* (NM_003482.4) transcripts were utilized in this analysis including nonsense, frameshift, missense, synonymous, deep intronic, and UTR variants. Multi‐allelic variants (homozygotes and hemizygotes) and structural variants (large deletions, duplications, insertions, or other DNA rearrangements) were excluded since they were not available in gnomAD v3.1 at the time of this analysis. Version 2.1 does include structural variant data; however, all variants were small deep intronic deletions, duplications, or insertions. Therefore, they did not seem relevant to this analysis. The gnomAD datasets provided sequence ontology, ClinVar significance, allele counts, allele frequencies, and chromosomal positions important for variant interpretation. ANOVAR22 was utilized to merge bioinformatic pathogenicity predictions and provided American College of Medical Genomics and American Molecular Pathology (ACMG‐AMP) criteria to further differentiate hotspot locations using pathogenic moderate (PM1). UCSC Genome Browser (GRCh38/hg38) was used to determine the genomic location of pathogenic and likely pathogenic variants when necessary.

### 2.2 Manual variant classification system

**FIGURE 1:**
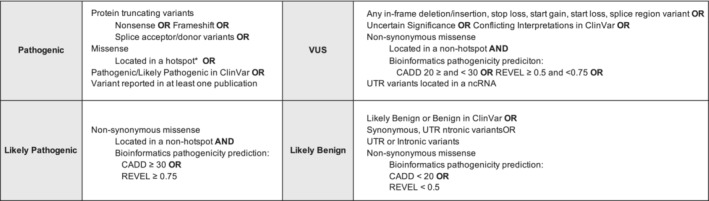
Manual genomic variant interpretation schematic. *Missense mutational hotspot locations were identified from the agreement in 2 conservation in silico models and a prior classification of the ACMG PM1 criteria. This variant interpretation schematic was adapted from Kim et al.^31^

A schematic (Figure 1) to classify all variants within *KMT2A‐D* as pathogenic (P), likely pathogenic (LP), variant of uncertain significance (VUS), and likely benign (LB) was modeled after the guidelines proposed by ACMG and a corresponding study that performed a similar analysis in *DICER1*.^31^ In brief, a variant was determined to be pathogenic if indicated as a loss of function (LOF) including nonsense or frameshift, a splice donor or acceptor variant, (a missense variant located in a missense mutational hotspot, reported in at least one publication, or had a Pathogenic/Likely Pathogenic call from ClinVar. Non‐synonymous missense variants located in a non‐hotspot region and having a bioinformatic pathogenicity prediction of CADD ≥30 or REVEL ≥0.75 were classified as likely pathogenic.

Our scheme deviates from Kim et al.'s^31^ classification system most notably in thresholds for bioinformatic pathogenicity predictor scores. Variants with CADD scores over 30 or REVEL scores over 0.75 are predicted to be the 0.1% most deleterious possible substitutions while variants with an intermediate CADD score of 20–29 and REVEL score of 0.5–0.74 are predicted to be the most 1% deleterious.^32^, ^33^ Thus, where Kim et al.^31^ enforced more conservative thresholds, we employed intermediate limits to further differentiate between missense variant classifications. However, a larger emphasis of their analysis was to look for solo variants with large effect sizes while ours was more liberal in allowing for moderate effect size variants that could potentially result in a phenotype if in conjunction with certain somatic variants. MetaSVM was not included in this paper due to significant inflation of missense variants classified as likely pathogenic across all four genes (*n* = 1446) compared to REVEL (*n* = 117) and CADD (*n* = 110), indicating it to be a possible artifact of the analysis, and recent publications also eliminating MetaSVM from its analysis while using similar CADD and REVEL thresholds.^34^

Identification of missense mutational hotspots

To date, there are no reported missense mutational hotspots within the *KMT2* genes except for the first PHD finger domain in *KMT2C*.^3^ To identify remaining hotspots, the PM1 ACMG criteria and mammalian conservation *in silico* models (GERP and PhyloP) were utilized. PM1 is a functional data criterion that is invoked when a variant is in a mutational hotspot and/or critical and well‐established functional domain (i.e., active site of an enzyme) without benign variation. Therefore, to confirm this as an identifier for missense variants being in a hotspot, variants additionally needed to be found within a conserved region in mammals and have a criterion of PM1 invoked.

### 2.3 ACMG classification

ACMG has previously developed guidance for the interpretation of clinical sequence variants.^35^ These recommendations primarily apply to the breadth of genetic tests used in clinical laboratories including genotyping single genes, panels, exomes, and genomes. For clinical relevance, the variants identified in gnomAD were also run through an ACMG auto‐classification system interpreting the *KMT2* variants in accordance with this criterion using Golden Helix VarSeq 2.2.0 (Golden Helix, Inc., Bozeman, MT) to annotate the datasets via the VSClinical pipeline. We note that due to the unavailability of parental samples for the gnomAD population, we did not include the ACMG PS2 or PM6 criteria for evidence of pathogenicity as part of our ACMG classification. To allow for further comparison with the manual variant classification system, the ACMG interpretations were classified into similar four categories of: LB, VUS, LP, and P. All variants that ACMG classified as benign or likely benign were combined into LB; all conflicting calls or variants of unknown significance were categorized as VUS including VUS/weak benign and VUS/weak pathogenic; all likely pathogenic were pooled as LP; all pathogenic variants were classified as pathogenic (P). Sex information was also collected from this pipeline and merged with our gnomAD dataset. Sex information was missing for 6423 variants (1225 for *KMT2A*, 1258 for *KMT2B*, 2036 for *KMT2C*, and 1904 for *KMT2D*), and was manually added for any missing P/LP variants. Total chromosomes/individuals and number of P/LP variants were used for prevalence calculations.

### 2.4 Data visualization and statistical analysis

Lollipop plots were created to display the spectrum of pathogenic and likely pathogenic variants across both interpretation schemes utilizing the cBioPortal Mutation Mapper.^36^ Variants classified as either pathogenic or likely pathogenic from the manual approach and pathogenic, likely pathogenic, and VUS/weak pathogenic from the ACMG interpretation were visualized in the lollipop plots for all four genes. Only protein‐coding variants were included, removing eight canonical splice variants (*KMT2C* = 5, *KMT2D* = 3). Logistic regression was used for analyzing ancestry and gender allele frequency differences across variant type and interpretation. All statistical analysis was done in R (4.1.2) and utilizing the package *epiDisplay*. The overall allele frequency for each gene was calculated by dividing the total allele count by the total allele number and was similar for the ancestry‐specific frequencies. Variant prevalence was calculated by dividing the amount of pathogenic or likely pathogenic variants by the total amount of individuals (Table S1). P‐values were only calculated for male/female mean allele frequencies comparison, a two‐sample *t*‐test was used to determine significance.

## 3 RESULTS

### 3.1 Classification of unique KMT2 variants from gnomAD

**FIGURE 2:**
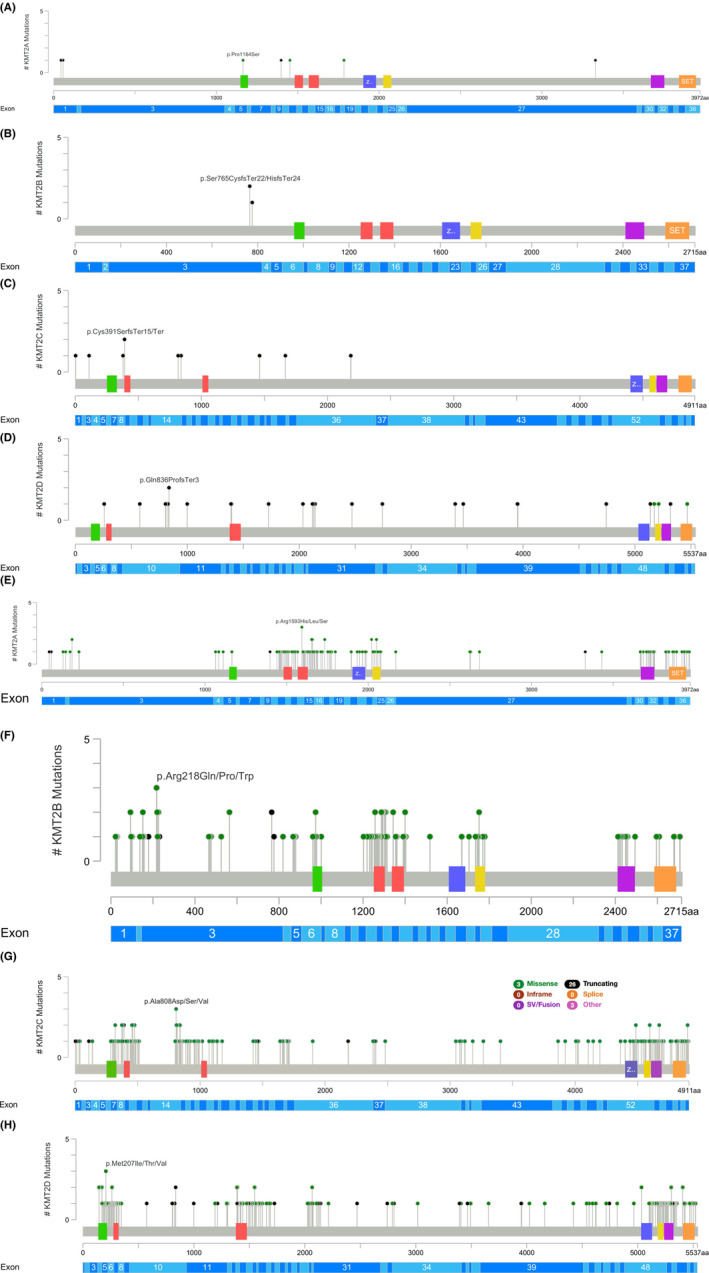
(A–G) Lollipop plots displaying amnio acid and exonic location of pathogenic and likely pathogenic *KMT2A‐D* variants across both interpretation methods. (A) KMT2A ACMG P/LP variants. (B) KMT2B ACMG P/LP variants. (C) KMT2C ACMG P/LP variants. Five splice site variants not shown (D) KMT2D ACMG P/LP variants. (E) KMT2A Manual P/LP variants. (F) KMT2B Manual P/LP variants. (G) KMT2C Manual P/LP variants. (H) KMT2D Manual P/LP variants. Black dots = Truncating variants (nonsense or frameshift). Green dots = Missense or splice variants. *p*‐values only computed for male/female allele frequency comparisons.

A total of 14,313 variants were analyzed from the gnomAD database (*KMT2A* = 2393, *KMT2B* = 2994, *KMT2C* = 4325, and *KMT2D* = 4601). Across all four genes, there were a total of 38 frameshifts, 17 nonsense (stop gained), 1 stop lost, 18 canonical splice sites (splice acceptor and splice donor), 509 non‐canonical splice site (>2 and < 10 base pairs from intron/exon boundary) 5558 missense, 248 small indels (in‐frame deletions and insertions), 172 5′ and 3′ UTR, 2961 synonymous, and 4791 intronic variants (Tables S2 and S3). Variants classified as pathogenic or likely pathogenic (including missense, nonsense, frameshift, and canonical splice site variants only) were further utilized for downstream analysis and stratified by ancestry. *KMT2C* (AF = 0.011, 0.001) and *KMT2D* (AF = 0.003, 0.0003) both had higher pathogenic and likely pathogenic allele frequencies across both interpretations (manual approach and ACMG, respectively) than *KMT2A* (AF = 0.002, 0.00005) and *KMT2B* (AF = 0.003, 0.00002). However, the manual approach consistently had higher frequencies when compared to the ACMG method (Table 1; Tables S3 and S4). All ACMG and manual pathogenic and likely pathogenic variants were plotted to highlight the amino acid and exonic locations of these variants (Figure 2A–G).

**TABLE 1:** *KMT2A‐D* total pathogenic/likely pathogenic variant prevalence and frequencies across ancestry and sex assigned at birth

		Overall				African American		Latino/Admixed American		East Asian		South Asian		European (non‐Finnish)		Ashkenazi Jewish		Finnish		Amish		Middle Eastern		Other		Male	Female	*p*‐value
		N	AC	AF	Prevalence	AC	AF	AC	AF	AC	AF	AC	AF	AC	AF	AC	AF	AC	AF	AC	AF	AC	AF	AC	AF	AF	AF	
Manual	KMT2A	129	290	1.96E‐03	1/255	90	2.19E‐03	11	7.28E‐04	13	2.61E‐03	16	3.33E‐03	126	1.94E‐03	4	1.21E‐03	27	2.54E‐03	0	0	0	0	2	9.88E‐04	7.28E‐03	4.85E‐03	0.06
	KMT2B	129	395	2.67E‐03	1/187	180	4.37E‐03	52	3.44E‐03	12	2.41E‐03	10	2.08E‐03	104	1.60E‐03	1	3.03E‐04	7	6.58E‐04	22	2.41E‐02	0	0	7	3.46E‐03	1.19E‐02	5.59E‐02	0.08
	KMT2C	219	1559	1.05E‐02	1/47	530	1.29E‐02	110	7.28E‐03	166	3.34E‐02	87	1.81E‐02	514	7.93E‐03	29	8.78E‐03	51	4.80E‐03	45	4.93E‐02	3	9.87E‐03	24	1.19E‐02	4.80E‐03	3.29E‐03	0.26
	KMT2D	183	458	3.09E‐03	1/161	89	2.16E‐03	71	4.70E‐03	24	4.83E‐03	25	1.70E‐04	217	3.35E‐03	16	4.85E‐03	3	2.82E‐04	1	1.10E‐03	1	3.29E‐03	11	5.43E‐03	3.03E‐04	1.70E‐04	0.1
ACMG	KMT2A	7	7	4.73E‐05	1/10574	0	0	0	0	0	0	1	2.08E‐04	5	7.71E‐05	0	0	1	9.41E‐05	0	0	0	0	0	0	2.08E‐04	8.78E‐03	0.03
	KMT2B	3	3	2.03E‐05	1/24674	0	0	0	0	3	6.03E‐04	0	0	0	0	0	0	0	0	0	0	0	0	0	0	1.70E‐04	1.70E‐04	0.86
	KMT2C	19	145	9.79E‐04	1/3896	51	1.24E‐03	7	4.63E‐04	0	0	4	8.32E‐04	24	3.70E‐04	1	3.03E‐04	11	1.03E‐03	51	5.59E‐02	1	3.29E‐03	3	1.48E‐03	4.63E‐04	5.20E‐03	0.54
	KMT2D	32	41	2.77E‐04	1/2314	12	2.92E‐04	5	3.31E‐04	4	8.05E‐04	8	1.66E‐03	11	1.70E‐04	0	0	0	0	1	1.10E‐03	0	0	0	0	1.24E‐03	1.70E‐04	0.43

Abbreviations: AC, Allele count; AF, Allele frequency; N, Number of P/LP variants.

### 3.2 Manual Variant Interpretation

The manual variant classification system identified 534 pathogenic variants (*KMT2A* = 109, *KMT2B* = 119, *KMT2C* = 167, *KMT2D* = 139) and 126 likely pathogenic variants (*KMT2A* = 20, *KMT2B* = 10, *KMT2C* = 52, *KMT2D* = 44) (Table S2). There were additionally 3837 variants of uncertain significance identified and 9816 likely benign (Table S2). In total, 461 missense variants, 38 frameshift variants, 17 nonsense, and 18 canonical splice site variants were identified as pathogenic (Table S2). All 126 likely pathogenic variants are non‐synonymous missense per the schematic (Figure 1).

### 3.3 ACMG criteria

Classifying variants according to ACMG criteria revealed five pathogenic variants: 1 frameshift variant in *KMT2A* (1 allele) along with 3 frameshifts (3 alleles) and 1 nonsense variant (1 allele) in *KMT2D*. A total of 56 likely pathogenic variants were identified (*KMT2A* = 6, *KMT2B* = 3, *KMT2C* = 19, *KMT2D* = 28) consisting of 8 canonical splice sites, 33 frameshifts, 9 nonsense, and 6 missense variants (Table S3). 5735 variants of uncertain significance and 8517 likely benign variants were also identified (Table S3). Of note, there were no pathogenic variants identified in *KMT2B* or *KMT2C* (Table S3).

### 3.4 Intronic and UTR variants

In the manual variant interpretation scheme, a total of 8835 likely benign intronic variants were excluded from our analysis bringing the total sample variants to 4791 utilized. Likewise, in the ACMG criteria schematic, a total of 8840 likely benign intronic variants were excluded. A comparison of interpretation methods reveals minor differences between them for both intronic and UTR variants. In *KMT2A* and *KMT2C*, the manual approach identified no intronic variants as being variants of unknown significance; however, 5 for *KMT2A* and 14 for *KMT2C* were noted in the ACMG method. *KMT2B* and *KMT2D* had intronic variants identified as VUS in the manual approach (*n* = 5 and *n* = 8) which was reflected in the ACMG criteria by identifying 11 and 9, respectively. In terms of UTR variants, the manual approach had 15 denoted as VUS for *KMT2A* due to their designation as ncRNA while the ACMG criteria retained only 5 of those with the other 10 reclassified as likely benign. For *KMT2B*, *KMT2C*, and *KMT2D*, none were classified above likely benign in the manual classification; however, in the ACMG criteria, these genes had UTR variants classified as VUS (*KMT2B* = 2, *KMT2C* = 9, *KMT2D* = 2) (Tables S2 and S3).

### 3.5 Ancestry and sex‐based differences in pathogenic and likely pathogenic variants

There were multiple ancestry‐specific variants identified in this analysis (Table 1; Tables S4 and S5). Of the four frameshift variants (4 alleles) classified as pathogenic by the manual approach for *KMT2A*, one (c.9975_9981del, p.Leu3329GlnfsTer14) was from the South Asian population (1 allele, AF = 2.08E‐04) and another (c.134del, p.Pro45ArgfsTer105) from the Finnish population (1 allele, AF = 9.41E‐05). Regarding ACMG criteria, the South Asian variant was additionally one of only three frameshift variants identified as likely pathogenic and the Finnish frameshift variant was the only pathogenic variant identified according to this approach. Further, an Ashkenazi Jewish variant (c.3490C > T, p.Pro1164Ser) was one of three missense variants classified as likely pathogenic by ACMG (1 allele, AF = 3.03E‐04). In *KMT2B*, of the two nonsense variants denoted as pathogenic according to the manual classification system (3 alleles), one (c.536C > G, p.Ser179Ter) was from the Latino/Admixed American population (2 alleles, AF = 1.32E‐04) and another (c.692C > G, p.Ser231Ter) from the South Asian population (1 allele, AF = 2.08E‐04). Additionally, three of the four frameshift variants identified as pathogenic by the manual approach were found to be from the East Asian population (c.2292del, p.Ser765HisfsTer24; c.2294_2300del, p.Ser765CysfsTer22; c.2326_2327insCT, p.Arg776ProfsTer14) each with 1 allele count and total AF = 6.03E‐04. In the Amish population, one variant in *KMT2B* (c.412C > T, p.Arg138Cys) with an AF = 2.41E‐02 contributed to 22 out of the 39 total alleles for variants classified as likely pathogenic by the manual approach. Further, an Amish variant in *KMT2C* (c.7443‐2del) with an AF = 4.61E‐02 contributed to 42 out of 69 total alleles for all likely pathogenic canonical splice site variants according to ACMG. *KMT2C* also saw one variant from the African American population (c.1173C > A, p.Cys391Ter) account for 31 of 69 total alleles for nonsense variants identified as pathogenic by the manual classification system (AF = 7.53E‐04) which had a similar effect on nonsense variants classified as likely pathogenic by ACMG. Likewise, one African American variant (c.7443‐2dup) had 143 alleles out of 384 total for pathogenic canonical splice site variants according to the manual approach. Lastly, regarding *KMT2D* classification according to ACMG ‐AMP criteria, the only nonsense variant (1 allele, AF = 2.43E‐05) identified as pathogenic was from the African American population (c.7411C > T, p.Arg2471Ter), one out of three frameshift variants classified as pathogenic was from the East Asia population (c.2994del, p.Met999Ter), and two of the three likely pathogenic canonical splice site variants were from the South Asian population (c.14252‐1G > A; c.5533 + 2 T > C).

For those with data on sex assigned at birth, according to the manual classification system, males tended to have higher average allele frequencies than females, except for *KMT2B* (Table 1). Information was missing for nearly all ACMG criteria except for likely pathogenic variants in *KMT2C* leading to an average AF for males of 2.64E‐06 and 2.06E‐06 for females along with one pathogenic frameshift variant for *KMT2D* (c.4168dup, p.Ala1390GlyfsTer42) one male allele (AF = 6.01E‐09) and none for females (Table 1).

## 4 DISCUSSION

Since the relationship between *KMT2A* and both myeloid and lymphoid leukemias was first discovered, its crucial role as an epigenetic regulator has been further characterized along with the importance of other gene family members.^2^, ^37^ Of recent interest has been their relationship with IL. While structural rearrangements of *KMT2A* have long been recognized as a key feature of this rare cancer, many components regarding the etiology of IL remain unknown. Given the inability to fully account for the incidence of IL under somatic variant mechanisms, studies now suggest this complex trait results from many rare inherited variants that, in aggregate, influence disease phenotypes.^22^, ^26^, ^38^ It has been further complicated by the failure of murine models (in *KMT2A* rearrangements) to develop leukemia in infancy, suggesting other factors are necessary for rapid‐onset leukemia *in utero*.^39^ Therefore, while individual parents may not have significantly increased cancer risk, random combinations of alleles during offspring development can lead to a greater chance of leukemogenesis in infancy from an enhanced germline genetic predisposition.^26^ Indeed, one analysis reported a statistically significant increase in adjusted odds ratio when a second‐degree relative was confirmed to have cancer for infants with ALL (adj. OR = 1.66, 95% CI = 1.12–2.45) and near significant increase for AML (adj. OR = 1.54, 95% CI = 0.80–2.98).^40^, ^41^ Multiple *KMT2* genes have also now been shown to potentially play a role in the onset of leukemogenesis with the presence of excess congenital, non‐synonymous germline variation in *MLL‐3* (*KMT2C*) having been identified specifically.^26^ This study, therefore, further inquired upon the prevalence of germline pathogenic variation in *KMT2A‐D* in the general population.

An overall total of 14,313 variants were identified across all four *KMT2* genes (pathogenic, likely pathogenic, VUS, and likely benign). The manual approach classified variants as pathogenic or likely pathogenic 4.61% of the time compared to 0.43% according to ACMG. Five variants identified as pathogenic by both approaches included one *KMT2A* frameshift variant (c.134del, p.Pro45ArgfsTer105) as well as three *KMT2D* frameshifts (c.15953_15956del, p.Leu5318SerfsTer14; c.4168dup, p.Ala1390GlyfsTer42; and c.2994del, p.Met999Ter) and one nonsense (c.7411C > T, p.Arg2471Ter). Both c.7411C > T and c.2994del along with two other *KMT2D* variants identified as pathogenic and likely pathogenic by ACMG criteria (c.4168dup, p.A1390GfsTer42 and c.10180C > T, p.Gln3394Ter) have been reported to be *de novo* in individuals affected with Kabuki syndrome.^29^, ^42^, ^43^, ^44^, ^45^, ^46^ Given the autosomal dominant inheritance pattern, this brings into question why damaging germline variants would be present among individuals or any first‐degree relative screened for severe pediatric disease within the gnomAD database.^46^ Penetrance is considered nearly complete for *KMT2D*; therefore, it is likely these individuals may have mosaic Kabuki, which has been found in two studies to result in individuals that are mildly/minimally affected.^29^, ^47^, ^48^

An increased number of damaging variant classifications from the manual approach largely resulted from its automatic classification of missense variants located in a missense mutational hotspot as pathogenic and broad categorizations based on variant type more generally (Figure 1). Further, there was an enrichment of likely pathogenic missense variants given their classification based solely on bioinformatic *in silico* predictor scores (CADD ≥30 and REVEL ≥0.75). Besides missense variants, those downgraded from pathogenic by ACMG to VUS or likely benign included: canonical splice site variants (*KMT2A* = 2, *KMT2C* = 2, *KMT2D* = 6), nonsense variants (*KMT2B* = 2, *KMT2C* = 2, *KMT2D* = 3), and one frameshift variant for *KMT2B*. No variants classified as likely benign or VUS in the manual variant classification system were reclassified as pathogenic or likely pathogenic by ACMG. Overall, the ACMG method was significantly more conservative across all four genes. Such results were expected given ACMG is the current clinical standard of genetic variant interpretation, thus requiring additional criteria to classify a variant as pathogenic or likely pathogenic. Our findings indicate that approaches created to aid in population genetic research may lead to higher estimates of damaging variants, particularly for novel genes and complex diseases.

*KMT2*‐family variants are among the most frequent somatic alterations in human cancer.^2^ *KMT2A* rearrangements represent recurrent somatic events comprising 35%–50% of infant AML cases^24^, ^25^ and 50%–80% of infant ALL cases.^24^, ^49^ Four germline variants identified in our analysis of *KMT2C* (c.1173C > A and c.2976 + 1G > C) and *KMT2D* (c.2560dup and c.4168dup) were also found in COSMIC (the Catalogue Of Somatic Mutations In Cancer) in hematopoietic and lymphoid tumors. All four of these variants were classified as likely pathogenic or pathogenic in both the manual and ACMG interpretations. Overall, the frequency of germline pathogenic variants that were found in the somatic literature was low. This illustrates the growing importance of understanding the background frequency of *KMT2* germline variants in large, cancer‐free populations.

The use of the gnomAD database to characterize the variant spectrum of these four genes offered numerous benefits including access to large sample sizes and ancestry‐specific data. This allowed for a closer look at allele frequencies by ancestry for pathogenic and likely pathogenic variants (Table 1; Tables S4 and S5). GnomAD v3 included individuals from Amish and Middle Eastern populations leading to interesting results. For instance, out of the 10 likely pathogenic missense variants classified by the manual classification system for *KMT2B*, a single missense variant was found in 39 alleles (c.412C > T, p.Arg138Cys), 56% of those alleles were of Amish ancestry (AF = 2.41E‐02) suggesting a possible founder mutation (Table S4). Females tended to have lower allele frequencies for pathogenic and likely pathogenic variants compared to males (Table 1). Such findings were unexpected given previous studies showing infants with leukemia are more often female and odds ratios significantly increasing when a child's father previously had cancer (adj. OR = 2.34, 95% CI = 0.96–5.77 for AML; adj. OR = 1.80, 95% CI = 1.04–3.12 for ALL).^16^, ^41^

The widespread availability of population‐based exome and genome sequencing has prompted a revision in our current understanding of tumor‐predisposition genes. According to our manual method, P/LP variants were more prevalent (0.39%–2.13%, 1/47–1/255), than what was identified in ACMG (0.004–0.04%, 1/2314–1/24674) (Table 1). Aligned with this observation, there are much more non‐penetrant, pathogenic variation in BRCA1/2 (0.2%–0.33%, 1/300–1/500),^50^ TP53 (0.2%, 1/500)^30^ and mismatch repair genes (Lynch syndrome; 0.23%,1/440)^51^ than previously expected.^30^ Additionally, there are well‐known, highly penetrant genetic disorders with population frequencies similar to our *KMT2A‐D* pathogenic variation estimates (0.004%–2.13%), including velo‐cardio‐facial syndrome (0.05%, 1/2000),^52^ cystic fibrosis (0.04%,1/2500 in white Americans/Europeans),^53^ neurofibromatosis type 1 (0.03%,1/3000),^54^ Duchenne muscular dystrophy (0.01%–0.02%,1/5000–1/7500 in males),^55^ Williams syndrome (0.013%, 1/7500),^56^ and Marfan syndrome (0.01%–0.013%,1/7500–1/10,000).^57^ A broader representation of the frequency of these variants in the general population helps our understanding of the penetrance and the true population frequency.

Overall, this approach allowed for an examination of germline variation within the *KMT2* genes and adds to the body of research on the significance of inherited germline heterozygous variants. However, there were several limitations. The manual interpretation includes a heavy reliance on variant type, *in silico* models, and published literature for determining variant classification. Therefore, it has limited usage in a clinical setting and is better suited for large‐scale epidemiological variant interpretation to reveal potential variations of interest for future investigation, as we have done herein. ClinVar is driven by submissions of data, and its scope is limited to variants that have been interpreted for clinical or functional significance, which is laborious for each variant. This is reflected here as ClinVar was considerably more conservative in identifying P/LP variants than the manual method. GnomAD does not provide individual genotype data; consequently, there is a possibility of one person possessing multiple P/LP *KMT2A‐D* variants. Additionally, the sample size of each ancestry population was not equivalent and varied greatly between ancestries. This complicated our ability to discern whether the lack of variants identified in the smaller (<5000 adults) ancestry populations (Middle Eastern, Amish, Other, Ashkenazi Jewish, South Asian, and East Asian) are due to a true absence or are due to a lack of statistical power to detect such variants. We were unable to control for the digenic or polygenic inheritance of the variants meaning the measures of frequencies used here, assumed that each variant was found in a unique person. We did not have parental genetic information; therefore, we could not differentiate between inherited or *de novo* germline variants and ACMG criteria PS2 was unable to be invoked to determine paternal or maternal inheritance. Lastly, gnomAD captures a picture of the cancer‐free adult population (18–85 years) with no personal or first‐degree family history of severe pediatric disease. However, it still may include individuals that possess other risk variants for polygenic, multifactorial, or adult‐onset conditions.

Publicly available population databases include whole genome and exome sequence information from large populations, making them useful in the establishment of a comprehensive resource on human genetic variation at the population level. They can be used to estimate the full spectrum of natural and human disease variation and are utilized in the ACMG criteria for classifying clinical variants.^35^ We presented a comprehensive characterization of *KMT2A*, *KMT2B*, *KMT2C*, and *KMT2D* pathogenic variation in a large cancer‐free adult population, with ancestry‐ and sex‐specific frequencies. Epidemiological genomic variant interpretation is often a bottleneck in population genomics analysis and is needed to understand gene penetrance, clinical genotype–phenotype relationships, and the prevalence of nonsynonymous variation. The findings from our study, if validated in other large, population‐based datasets (i.e., LOVD, UK BioBank), suggest that pathogenic heterozygous germline *KMT2A‐D* variants are more common than previously expected and provide better insight into the penetrance of these variants.

## AUTHOR CONTRIBUTIONS

**Jenna K. Larson:** Conceptualization (equal); data curation (equal); formal analysis (equal); methodology (equal); visualization (equal); writing – original draft (equal); writing – review and editing (equal). **DeVon N. Hunter‐Schlichting:** Conceptualization (equal); data curation (equal); formal analysis (equal); methodology (equal); project administration (equal); validation (equal); visualization (equal); writing – original draft (equal); writing – review and editing (equal). **Erin L. Crowgey:** Conceptualization (equal); formal analysis (equal); investigation (equal); project administration (equal); supervision (equal); writing – review and editing (equal). **Lauren J. Mills:** Conceptualization (equal); data curation (equal); methodology (equal); resources (equal); software (equal); supervision (equal); validation (equal); writing – review and editing (equal). **Todd E. Druley:** Conceptualization (equal); investigation (equal); project administration (equal); resources (equal); writing – review and editing (equal). **Erin Marcotte:** Conceptualization (equal); formal analysis (equal); funding acquisition (equal); investigation (equal); project administration (equal); supervision (equal); writing – original draft (equal); writing – review and editing (equal).

## CONFLICT OF INTEREST

TED is the Chief Medical Officer for Mission Bio, but Mission Bio had no role in the generation, analysis, or interpretation of these data. The authors declare no other competing interests.

## Supporting information


Table S1.
Table S2.
Table S3.
Table S4.
Table S5.

## Data Availability

The data that support the findings of this study are openly available in gnomAD at https://gnomad.broadinstitute.org/.

## References

[1] Li Y , Han J , Zhang Y , et al. Structural basis for activity regulation of MLL family methyltransferases. Nature. 2016;530(7591):447‐452. doi:10.1038/nature16952 26886794 PMC5125619

[2] Rao RC , Dou Y . Hijacked in cancer: the KMT2 (MLL) family of methyltransferases. Nat Rev Cancer. 2015;15:334‐346. doi:10.1038/nrc3929 25998713 PMC4493861

[3] Fagan RJ , Dingwall AK . COMPASS ascending: emerging clues regarding the roles of MLL3/KMT2C and MLL2/KMT2D proteins in cancer. Cancer Lett. 2019;458:56‐65. doi:10.1016/j.canlet.2019.05.024 31128216 PMC6638576

[4] Zhang P , Huang Y . Genomic alterations in KMT2 family predict outcome of immune checkpoint therapy in multiple cancers. J Hematol Oncol. 2021;14(1):1‐5. doi:10.1186/S13045-021-01050-0/FIGURES/2 33653367 PMC7927217

[5] Molin AD , Bresolin S , Gaffo E , et al. CircRNAs are here to stay: a perspective on the MLL recombinome. Front Genet. 2019;10(88). doi:10.3389/FGENE.2019.00088 PMC6382020 30815012

[6] O'Meara E , Stack D , Phelan S , et al. Identification of an MLL4‐GPS2 fusion as an oncogenic driver of undifferentiated spindle cell sarcoma in a child. Genes Chromosomes Cancer. 2014;53(12):991‐998. doi:10.1002/GCC.22208 25139254

[7] Saigo K , Yoshida K , Ikeda R , et al. Integration of hepatitis B virus DNA into the myeloid/lymphoid or mixed‐lineage leukemia (MLL4) gene and rearrangements of MLL4 in human hepatocellular carcinoma. Hum Mutat. 2008;29(5):703‐708. doi:10.1002/HUMU.20701 18320596

[8] Meeks JJ , Shilatifard A . Multiple roles for the MLL/COMPASS family in the epigenetic regulation of gene expression and in cancer. Ann Rev Cancer Biol. 2017;1:425‐446. doi:10.1146/ANNUREV-CANCERBIO-050216-034333

[9] Ford DJ , Dingwall AK . The cancer COMPASS: navigating the functions of MLL complexes in cancer. Cancer Genet. 2015;208(5):178‐191. doi:10.1016/J.CANCERGEN.2015.01.005 25794446

[10] di Fede E , Massa V , Augello B , et al. Expanding the phenotype associated to KMT2A variants: overlapping clinical signs between Wiedemann–Steiner and Rubinstein–Taybi syndromes. Eur J Hum Genet. 2020;29(1):88‐98. doi:10.1038/s41431-020-0679-8 32641752 PMC7852672

[11] Meyer E , Carss KJ , Rankin J , et al. Mutations in the histone methyltransferase gene KMT2B cause complex early‐onset dystonia. Nat Genet. 2016;49(2):223‐237. doi:10.1038/ng.3740 27992417

[12] Kleefstra T , Kramer JM , Neveling K , et al. Disruption of an EHMT1‐associated chromatin‐modification module causes intellectual disability. Am J Hum Genet. 2012;91(1):73‐82. doi:10.1016/J.AJHG.2012.05.003 22726846 PMC3397275

[13] Kleefstra T , Smidt M , Banning MJG , et al. Disruption of the gene Euchromatin Histone Methyl Transferase1 (Eu‐HMTase1) is associated with the 9q34 subtelomeric deletion syndrome. J Med Genet. 2005;42(4):299‐306. doi:10.1136/JMG.2004.028464 15805155 PMC1736026

[14] Lintas C , Persico AM . Unraveling molecular pathways shared by Kabuki and Kabuki‐like syndromes. Clin Genet. 2018;94(3–4):283‐295. doi:10.1111/CGE.12983 28139835

[15] Calvo C , Fenneteau O , Leverger G , Petit A , Baruchel A , Méchinaud F . Infant acute myeloid leukemia: a unique clinical and biological entity. Cancer. 2021;13(4):1‐14. doi:10.3390/CANCERS13040777 PMC7918235 33668444

[16] Marcotte EL , Druley TE , Johnson KJ , et al. Parental age and risk of infant leukemia: a pooled analysis. Paediatr Perinat Epidemiol. 2017;31(6):563‐572. doi:10.1111/ppe.12412 28940632 PMC5901723

[17] Linabery AM , Ross JA . Childhood and adolescent cancer survival in the U.S. by race and ethnicity (diagnostic period 1975–1999). Cancer. 2008;113(9):2575‐2596. doi:10.1002/CNCR.23866 18837040 PMC2765225

[18] Goldsby RE , Liu Q , Nathan PC , et al. Late‐occurring neurologic sequelae in adult survivors of childhood acute lymphoblastic leukemia: a report from the childhood cancer survivor study. J Clin Oncol. 2009;28:324‐331. doi:10.1200/JCO.2009.22.5060 19917844 PMC2815720

[19] Stefanski KJ , Anixt JS , Goodman P , et al. Long‐term neurocognitive and psychosocial outcomes after acute myeloid leukemia: a childhood cancer survivor study report. J Natl Cancer Inst. 2021;113(4):481‐495. doi:10.1093/JNCI/DJAA102 32797189 PMC8023820

[20] Wertheim G . Infant acute leukemia. Clin Lab Med. 2021;41(3):541‐550. doi:10.1016/j.cll.2021.04.002 34304781

[21] Gruhn B , Taub JW , Ge Y , et al. Prenatal origin of childhood acute lymphoblastic leukemia, association with birth weight and hyperdiploidy. Leukemia. 2008;22(9):1692‐1697. doi:10.1038/leu.2008.152 18548099

[22] Andersson AK , Ma J , Wang J , et al. The landscape of somatic mutations in Infant MLL rearranged acute lymphoblastic leukemias. Nat Genet. 2015;47(4):330‐337. doi:10.1038/NG.3230 25730765 PMC4553269

[23] Marcotte EL , Spector LG , Mendes‐de‐Almeida DP , Nelson HH . The prenatal origin of childhood leukemia: potential applications for epidemiology and newborn screening. Front Pediatr. 2021;9:188. doi:10.3389/FPED.2021.639479/BIBTEX PMC8102903 33968846

[24] Felix CA , Lange BJ . Leukemia in infants. Oncologist. 1999a;4(3):225‐240. doi:10.1634/THEONCOLOGIST.4-3-225 10394590

[25] Tomizawa D . Recent progress in the treatment of infant acute lymphoblastic leukemia. Pediatr Int. 2015;57(5):811‐819. doi:10.1111/PED.12758 26215843

[26] Valentine MC , Linabery AM , Chasnoff S , et al. Excess congenital non‐synonymous variation in leukemia‐associated genes in MLL‐ infant leukemia: a children's oncology group report. Leukemia. 2014;28(6):1235‐1241. doi:10.1038/leu.2013.367 24301523 PMC4045651

[27] Maurya S , Yang W , Tamai M , et al. Loss of KMT2C reprograms the epigenomic landscape in hPSCs resulting in NODAL overexpression and a failure of hemogenic endothelium specification. Epigenetics. 2021;17(2):220‐238. doi:10.1080/15592294.2021.1954780 34304711 PMC8865227

[28] Karczewski KJ , Francioli LC , Tiao G , et al. The mutational constraint spectrum quantified from variation in 141,456 humans. Nature. 2020;581:19‐443. doi:10.1038/s41586-020-2308-7 32461654 PMC7334197

[29] Adam MP , Banka S , Bjornsson HT , et al. Kabuki syndrome: international consensus diagnostic criteria. J Med Genet. 2019;56(2):89‐95. doi:10.1136/JMEDGENET-2018-105625 30514738

[30] Gröbner SN , Worst BC , Weischenfeldt J , et al. The landscape of genomic alterations across childhood cancers. Nature. 2018;555(7696):321‐327. doi:10.1038/NATURE25480 29489754

[31] Kim J , Field A , Schultz KAP , Hill DA , Stewart DR . The prevalence of DICER1 pathogenic variation in population databases. Int J Cancer. 2017;141(10):2030‐2036. doi:10.1002/IJC.30907 28748527 PMC5749397

[32] Ioannidis NM , Rothstein JH , Pejaver V , et al. REVEL: an ensemble method for predicting the pathogenicity of rare missense variants. Am J Hum Genet. 2016;99(4):877‐885. doi:10.1016/J.AJHG.2016.08.016 27666373 PMC5065685

[33] Kircher M , Witten DM , Jain P , O'Roak BJ , Cooper GM , Shendure J . A general framework for estimating the relative pathogenicity of human genetic variants. Nat Genet. 2014;46(3):310‐315. doi:10.1038/NG.2892 24487276 PMC3992975

[34] Mirshahi UL , Kim J , Best AF , et al. A genome‐first approach to characterize DICER1 pathogenic variant prevalence, penetrance, and phenotype. JAMA Netw Open. 2021;4(2):e210112. doi:10.1001/JAMANETWORKOPEN.2021.0112 33630087 PMC7907958

[35] Richards S , Aziz N , Bale S , et al. Standards and guidelines for the interpretation of sequence variants: a joint consensus recommendation of the American College of Medical Genetics and Genomics and the Association for Molecular Pathology. Genet Med. 2015a;17(5):405‐424. doi:10.1038/GIM.2015.30 25741868 PMC4544753

[36] Cerami E , Gao J , Dogrusoz U , et al. The cBio cancer genomics portal: an open platform for exploring multidimensional cancer genomics data. Cancer Discov. 2012;2(5):401‐404. doi:10.1158/2159-8290.CD-12-0095 22588877 PMC3956037

[37] Tkachuk DC , Kohler S , Cleary ML . Involvement of a homolog of Drosophila trithorax by 11q23 chromosomal translocations in acute leukemias. Cell. 1992;71(4):691‐700. doi:10.1016/0092-8674(92)90602-9 1423624

[38] Lupski JR , Belmont JW , Boerwinkle E , Gibbs RA . Clan genomics and the complex architecture of human disease. Cell. 2011;147(1):32‐43. doi:10.1016/J.CELL.2011.09.008 21962505 PMC3656718

[39] Bursen A , Schwabe K , Rü B , et al. The AF4MLL fusion protein is capable of inducing ALL in mice without requirement of MLLAF4. Blood. 2010;115(17):3570‐3579. doi:10.1182/blood-2009-06-229542 20194896

[40] Clarkson BD , Boyse EA . Possible explanation of the high concoddance for acute leukaemia in monozygotic twins. Lancet. 1971;297(7701):699‐701. doi:10.1016/S0140-6736(71)92705-X 4101637

[41] Couto AC , Ferreira JD , Koifman S , et al. Familial history of cancer and leukemia in children younger than 2 years of age in Brazil. Eur J Cancer Prev. 2013;22(2):151‐157. doi:10.2307/48504265 22926509

[42] Bögershausen N , Gatinois V , Riehmer V , et al. Mutation update for kabuki syndrome genes KMT2D and KDM6A and further delineation of X‐linked kabuki syndrome subtype 2. Hum Mutat. 2016;37(9):847‐864. doi:10.1002/HUMU.23026 27302555

[43] Digilio MC , Gnazzo M , Lepri F , et al. Congenital heart defects in molecularly proven Kabuki syndrome patients. Am J Med Genet A. 2017;173(11):2912‐2922. doi:10.1002/AJMG.A.38417 28884922

[44] Fokstuen S , Makrythanasis P , Hammar E , et al. Experience of a multidisciplinary task force with exome sequencing for Mendelian disorders. Hum Genomics. 2016;10(1):24. doi:10.1186/S40246-016-0080-4 27353043 PMC4924303

[45] Marangoni M , Smits G , Ceysens G , et al. Implementation of fetal clinical exome sequencing: Comparing prospective and retrospective cohorts. Genet Med. 2022;24(2):344‐363. doi:10.1016/J.GIM.2021.09.016 34906519

[46] Miyake N , Koshimizu E , Okamoto N , et al. MLL2 and KDM6A mutations in patients with Kabuki syndrome. Am J Med Genet. 2013;161(9):2234‐2243. doi:10.1002/AJMG.A.36072 23913813

[47] Banka S , Howard E , Bunstone S , et al. MLL2 mosaic mutations and intragenic deletion–duplications in patients with Kabuki syndrome. Clin Genet. 2013;83(5):467‐471. doi:10.1111/J.1399-0004.2012.01955.X 22901312

[48] Romana Lepri F , Cocciadiferro D , Augello B , et al. Clinical and neurobehavioral features of three novel kabuki syndrome patients with mosaic KMT2D mutations and a review of literature. Int J Mol Sci. 2017;19(1). doi:10.3390/ijms19010082 PMC5796032 29283410

[49] Greaves MF , Wiemels J . Origins of chromosome translocations in childhood leukaemia. Nat Rev Cancer. 2003;3(9):639‐649. 12951583 10.1038/nrc1164

[50] Chen S , Parmigiani G . Meta‐analysis of BRCA1 and BRCA2 penetrance. J Clin Oncol. 2007;25(11):1329‐1333. doi:10.1200/JCO.2006.09.1066 17416853 PMC2267287

[51] Rubenstein JH , Enns R , Heidelbaugh J , et al. American Gastroenterological Association Institute guideline on the diagnosis and management of lynch syndrome. Gastroenterology. 2015;149(3):777‐782. doi:10.1053/j.gastro.2015.07.036 26226577

[52] Shprintzen RJ . Velo‐cardio‐facial syndrome: 30 years of study. Dev Disabil Res Rev. 2008;14(1):3‐10. doi:10.1002/DDRR.2 18636631 PMC2805186

[53] Scotet V , Duguépéroux I , Saliou P , et al. Evidence for decline in the incidence of cystic fibrosis: a 35‐year observational study in Brittany, France. Orphanet J Rare Dis. 2012;7(1):1‐7. doi:10.1186/1750-1172-7-14/TABLES/4 22380742 PMC3310838

[54] Messiaen LM , Wimmer K . NF1 mutational spectrum. Monogr Hum Genet. 2008;16:63‐77. doi:10.1159/000126545

[55] Romitti PA , Zhu Y , Puzhankara S , et al. Prevalence of duchenne and becker muscular dystrophies in the United States. Pediatrics. 2015;135(3):513‐521. doi:10.1542/PEDS.2014-2044 25687144 PMC4477633

[56] Strømme P , Bjømstad PG , Ramstad K . Prevalence estimation of williams syndrome. J Child Neurol. 2016;17(4):269‐271. 10.1177/088307380201700406 12088082

[57] Groth KA , Hove H , Kyhl K , et al. Prevalence, incidence, and age at diagnosis in Marfan Syndrome Rare systemic diseases. Orphanet J Rare Dis. 2015;10(1):1‐10. doi:10.1186/S13023-015-0369-8/FIGURES/5 26631233 PMC4668669

