# Supplementary material for: KMT2A‐D pathogenicity, prevalence, and variation according to a population database
Source: Cancer Med. 2022 Dec 8;12(6):7234–45. doi: 10.1002/cam4.5443 (PMC10067056; doi:10.1002/cam4.5443)
Supplement: Supplementary file 1 — Table S1. Table S2. Table S3. Table S4. Table S5. [file CAM4-12-7234-s001.pdf]

KMT2A			KMT2B		
Ancestry	Individuals (n)	Allele Numbers	Ancestry	Individuals (n)	Allele Numbers
African American	20583	41166	African American	20580	41160
Amish	456	912	Amish	456	912
Latino/Admixed American	7553	15106	Latino/Admixed American	7553	15106
Ashkenazi Jewish	1651	3302	Ashkenazi Jewish	1651	3302
East Asian	2486	4972	East Asian	2486	4972
Finnish	5316	10632	Finnish	5315	10630
Middle Eastern	152	304	Middle Eastern	152	304
European (non-Finnish)	32411	64822	European (non-Finnish)	32411	64822
South Asian	2403	4806	South Asian	2403	4806
Other	1012	2024	Other	1012	2024
Total	74023	148046	Total	74019	148038

KMT2C			KMT2D		
Ancestry	Individuals (n)	Allele Numbers	Ancestry	Individuals (n)	Allele Numbers
African American	20583	41166	African American	20583	41166
Amish	456	912	Amish	456	912
Latino/Admixed American	7553	15106	Latino/Admixed American	7553	15106
Ashkenazi Jewish	1651	3302	Ashkenazi Jewish	1651	3302
East Asian	2486	4972	East Asian	2486	4972
Finnish	5316	10632	Finnish	5316	10632
Middle Eastern	152	304	Middle Eastern	152	304
European (non-Finnish)	32408	64822	European (non-Finnish)	32411	64822
South Asian	2403	4806	South Asian	2403	4806
Other	1012	2024	Other	1012	2024
Total	74020	148046	Total	74023	148046

KMT2A (n=2393)		
Likely Benign	**1632**	
	inframe deletion	1
	splice site	8
	UTR (5'/3')	18
	missense	302
	synonymous	492
	intronic	811
Variant of Uncertain Significance	**632**	
	synonymous	2
	inframe insertion	7
	UTR (5'/3')	15
	inframe deletion	19
	splice site	46
	missense	543
Likely Pathogenic	**20**	
	missense	20
Pathogenic	**109**	
	splice donor/acceptor	2
	frameshift	4
	missense	103

KMT2B (n=2994)		
Likely Benign	**2210**	
	UTR (5'/3')	42
	missense	414
	synonymous	701
	intronic	1053
Variant of Uncertain Significance	**655**	
	inframe insertion	13
	intronic	5
	inframe deletion	23
	splice donor/acceptor	110
	missense	504
Likely Pathogenic	**10**	
	missense	10
Pathogenic	**119**	
	nonsense	2
	frameshift	4
	missense	113

KMT2C (n=4325)		
Likely Benign	**3045**	
	UTR (5'/3')	69
	missense	583
	synonymous	765
	intronic	1628
Variant of Uncertain Significance	**1061**	
	stop lost	1
	inframe insertion	11
	inframe deletion	27
	splice site	215
	missense	807
Likely Pathogenic	**52**	
	missense	52
Pathogenic	**167**	
	splice donor/acceptor	7
	nonsense	7
	frameshift	9
	missense	144

KMT2D (n=4601)		
Likely Benign	**2929**	
	UTR (5'/3')	28
	missense	659
	synonymous	956
	intronic	1286
Variant of Uncertain Significance	**1489**	
	intronic	8
	synonymous	45
	inframe insertion	50
	inframe deletion	97
	splice site	130
	missense	1159
Likely Pathogenic	**44**	
	missense	44
Pathogenic	**139**	
	nonsense	8
	splice donor/acceptor	9
	frameshift	21
	missense	101

KMT2A (n=2393)		
Likely Benign	**1372**	
	inframe deletion	3
	inframe insertion	4
	UTR (5'/3')	28
	splice site	46
	missense	70
	synonymous	415
	intronic	806
Variant of Uncertain Significance	**1014**	
	canonical splice site	2
	inframe insertion	3
	intronic	5
	UTR (5'/3')	5
	splice site	8
	inframe deletion	17
	synonymous	79
	missense	895
Likely Pathogenic	**6**	
	frameshift	3
	missense	3
Pathogenic	**1**	
	frameshift	1
KMT2B **(n=2994)**		
Likely Benign	**1919**	
	inframe deletion	7
	inframe insertion	6
	UTR (5'/3')	20
	splice site	96
	missense	96
	synonymous	644
	intronic	1047
	frameshift	1
	nonsense	2
Variant of Uncertain Significance	**1072**	
	UTR (5'/3')	22
	inframe insertion	7
	intronic	11
	splice site	14
	synonymous	57
	inframe deletion	16
	missense	945
Likely Pathogenic	**3**	
	frameshift	3
Pathogenic	**0**	

KMT2C (n=4325)		
Likely Benign	**2700**	
	inframe deletion	1
	inframe insertion	1
	UTR (5' / 3')	60
	missense	151
	splice site	181
	synonymous	692
	intronic	1614
Variant of Uncertain Significance	**1606**	
	stop lost	1
	splice donor/acceptor	2
	nonsense	2
	UTR (5'/3')	9
	inframe insertion	10
	intronic	14
	inframe deletion	27
	splice site	34
	synonymous	73
	missense	1435
Likely Pathogenic	**19**	
	splice donor/acceptor	5
	nonsense	5
	frameshift	9
Pathogenic	**0**	
KMT2D **(n=4601)**		
Likely Benign	**2526**	
	inframe deletion	11
	UTR (5'/3')	16
	inframe insertion	18
	splice site	111
	missense	168
	synonymous	923
	intronic	1279
Variant of Uncertain Significance	**2043**	
	UTR (5'/3')	21
	nonsense	3
	splice donor/acceptor	6
	intronic	9
	splice site	18
	inframe insertion	32
	synonymous	78
	inframe deletion	86
	missense	1792
Likely Pathogenic	**28**	
	missense	3
	splice donor/acceptor	3
	nonsense	4
	frameshift	18
Pathogenic	**4**	
	nonsense	1
	frameshift	3

Gene	Variants	Overall				African American		Latino/Admixed American		East Asian		South Asian		European (non-Finnish)		Ashkenazi Jewish		Finnish		Amish		Middle Eastern		Other	
		Total variants	AC	AF	Prevalence	AC	AF	AC	AF	AC	AF	AC	AF	AC	AF	AC	AF	AC	AF	AC	AF	AC	AF	AC	AF
KMT2A	**Pathogenic**	**109**	**262**	1.77E-03	1/282	**85**	2.06E-03	**11**	7.28E-04	**10**	2.01E-03	**13**	2.70E-03	**114**	1.76E-03	**2**	6.06E-04	**24**	2.26E-03	**0**	0	**0**	0	**2**	9.88E-04
	missense	103	253	1.71E-03	3/877	83	2.02E-03	10	6.62E-04	10	2.01E-03	12	2.50E-03	111	1.71E-03	2	6.06E-04	23	2.16E-03	0	0	0	0	2	9.88E-04
	splice donor/acceptor	2	5	3.38E-05	1/14784	2	4.86E-05	1	6.62E-05	0	0	0	0	2	3.09E-05	0	0	0	0	0	0	0	0	0	0
	frameshift	4	4	2.70E-05	1/18480	0	0	0	0	0	0	1	2.08E-04	2	3.09E-05	0	0	1	9.41E-05	0	0	0	0	0	0
	**Likely Pathogenic**	**20**	**28**	1.89E-04	1/2640	**5**	1.21E-04	**0**	0	**3**	6.03E-04	**3**	6.24E-04	**12**	1.85E-04	**2**	6.06E-04	**3**	2.82E-04	**0**	0	**0**	0	**0**	0
	missense	20	28	1.89E-04	1/2640	5	1.21E-04	0	0	3	6.03E-04	3	6.24E-04	12	1.85E-04	2	6.06E-04	3	2.82E-04	0	0	0	0	0	0
KMT2B	**Pathogenic**	**119**	**356**	2.40E-03	1/208	**177**	4.30E-03	**52**	3.44E-03	**9**	1.81E-03	**9**	1.87E-03	**95**	1.47E-03	**1**	3.03E-04	**6**	5.64E-04	**0**	0	**0**	0	**7**	3.46E-03
	missense	113	349	2.36E-03	4/847	177	4.30E-03	50	3.31E-03	6	1.21E-03	8	1.66E-03	94	1.45E-03	1	3.03E-04	6	5.64E-04	0	0	0	0	7	3.46E-03
	nonsense	2	3	2.03E-05	1/24640	0	0	87	5.76E-03	0	0	1	2.08E-04	0	0	0	0	0	0	0	0	0	0	0	0
	frameshift	4	4	2.70E-05	1/18480	0	0	0	0	3	6.03E-04	0	0	1	1.54E-05	0	0	0	0	0	0	0	0	0	0
	**Likely Pathogenic**	**10**	**39**	2.63E-04	1/1895	**3**	7.29E-05	**0**	0	**3**	6.03E-04	**1**	2.08E-04	**9**	1.39E-04	**0**	0	**1**	9.41E-05	**22**	2.41E-02	**0**	0	**0**	0
	missense	10	39	2.63E-04	1/1895	3	7.29E-05	0	0	3	6.03E-04	1	2.08E-04	9	1.39E-04	0	0	1	9.41E-05	22	2.41E-02	0	0	0	0
KMT2C	**Pathogenic**	**167**	**1451**	9.80E-03	1/51	**484**	1.18E-02	**97**	6.42E-03	**162**	3.26E-02	**80**	1.66E-02	**484**	7.47E-03	**27**	8.18E-03	**45**	4.23E-03	**45**	4.93E-02	**3**	9.87E-03	**24**	1.19E-02
	missense	144	989	6.68E-03	1/75	290	7.04E-03	68	4.50E-03	97	1.95E-02	73	1.52E-02	402	6.20E-03	8	2.42E-03	33	3.10E-03	2	2.19E-03	2	6.58E-03	14	6.92E-03
	frameshift	9	9	6.08E-05	1/18480	4	9.72E-05	2	1.32E-04	0	0	0	0	1	1.54E-05	0	0	2	1.88E-04	0	0	0	0	0	0
	nonsense	7	69	4.66E-04	1/1071	31	7.53E-04	6	3.97E-04	0	0	4	8.32E-04	14	2.16E-04	1	3.03E-04	8	7.52E-04	1	1.10E-03	1	3.29E-03	3	1.48E-03
	splice donor/acceptor	7	384	2.59E-03	1/193	159	3.86E-03	21	1.39E-03	65	1.31E-02	3	6.24E-04	67	1.03E-03	18	5.45E-03	2	1.88E-04	42	4.61E-02	0	0	7	3.46E-03
	**Likely Pathogenic**	**52**	**108**	7.30E-04	1/684	**46**	1.12E-03	**13**	8.61E-04	**4**	8.05E-04	**7**	1.46E-03	**30**	4.63E-04	**2**	6.06E-04	**6**	5.64E-04	**0**	0	**0**	0	**0**	0
	missense	52	108	7.30E-04	1/684	46	1.12E-03	13	8.61E-04	4	8.05E-04	7	1.46E-03	30	4.63E-04	2	6.06E-04	6	5.64E-04	0	0	0	0	0	0
KMT2D	**Pathogenic**	**139**	**226**	1.53E-03	1/327	**63**	1.53E-03	**26**	1.72E-03	**20**	4.02E-03	**18**	3.75E-03	**84**	1.30E-03	**7**	2.12E-03	**3**	2.82E-04	**1**	1.10E-03	**1**	3.29E-03	**3**	1.48E-03
	missense	101	179	1.21E-03	1/413	49	1.19E-03	19	1.26E-03	15	3.02E-03	10	2.08E-03	74	1.14E-03	7	2.12E-03	1	9.41E-05	0	0	1	3.29E-03	3	1.48E-03
	frameshift	21	26	1.76E-04	1/2843	8	1.94E-04	3	1.99E-04	2	4.02E-04	5	1.04E-03	7	1.08E-04	0	0	0	0	1	1.10E-03	0	0	0	0
	nonsense	8	9	6.08E-05	1/18480	4	9.72E-05	2	1.32E-04	2	4.02E-04	0	0	1	1.54E-05	0	0	0	0	0	0	0	0	0	0
	splice donor/acceptor	9	12	8.11E-05	1/6160	2	4.86E-05	2	1.32E-04	1	2.01E-04	3	6.24E-04	2	3.09E-05	0	0	2	1.88E-04	0	0	0	0	0	0
	**Likely Pathogenic**	**44**	**232**	1.57E-03	1/319	**26**	6.32E-04	**45**	2.98E-03	**4**	8.05E-04	**7**	1.46E-03	**133**	2.05E-03	**9**	2.73E-03	**0**	0	**0**	0	**0**	0	**8**	3.95E-03
	missense	44	232	1.57E-03	1/319	26	6.32E-04	45	2.98E-03	4	8.05E-04	7	1.46E-03	133	2.05E-03	9	2.73E-03	0	0	0	0	0	0	8	3.95E-03

Gene	Variants	Overall				African American		Latino/Admixed American		East Asian		South Asian		European (non-Finnish)		Ashkenazi Jewish		Finnish		Amish		Middle Eastern		Other	
		Total variants	AC	AF	Prevalence	AC	AF	AC	AF	AC	AF	AC	AF	AC	AF	AC	AF	AC	AF	AC	AF	AC	AF	AC	AF
KMT2A	**Pathogenic**	**1**	**1**	**6.75E-06**	**1/74023**	**0**	**0**	**0**	**0**	**0**	**0**	**0**	**0**	**0**	**0**	**0**	**0**	**0**	**0**	**0**	**0**	**0**	**0**	**0**	**0**
	frameshift	1	1	6.75E-06	1/74023	0	0	0	0	0	0	0	0	0	0	0	0	1	9.41E-05	0	0	0	0	0	0
	**Likely Pathogenic**	**6**	**6**	**4.05E-05**	**1/12337**	**0**	**0**	**0**	**0**	**0**	**0**	**0**	**0**	**0**	**0**	**0**	**0**	**0**	**0**	**0**	**0**	**0**	**0**	**0**	**0**
	frameshift	3	3	2.03E-05	1/24674	0	0	0	0	0	0	1	2.08E-04	3	4.63E-05	0	0	0	0	0	0	0	0	0	0
	missense	3	3	2.03E-05	1/24674	0	0	0	0	0	0	0	0	2	3.09E-05	0	0	0	0	0	0	0	0	0	0
KMT2B	**Likely Pathogenic**	**3**	**3**	**2.03E-05**	**1/24674**	**0**	**0**	**0**	**0**	3	6.03E-04	**0**	**0**	**0**	**0**	**0**	**0**	**0**	**0**	**0**	**0**	**0**	**0**	**0**	**0**
	frameshift	3	3	2.03E-05	1/24674	0	0	0	0	3	6.03E-04	0	0	0	0	0	0	0	0	0	0	0	0	0	0
KMT2C	**Likely Pathogenic**	**19**	**145**	**9.79E-04**	**1/3896**	**51**	**1.24E-03**	**7**	**4.63E-04**	**0**	**0**	**4**	**8.32E-04**	**24**	**3.70E-04**	**1**	**3.03E-04**	**11**	**1.03E-03**	**51**	**5.59E-02**	**1**	**3.29E-03**	**3**	**1.48E-03**
	frameshift	9	9	6.08E-05	1/8225	4	9.72E-05	2	1.32E-04	0	0	0	0	1	1.54E-05	0	0	2	1.88E-04	0	0	0	0	0	0
	splice acceptor/donor	5	69	4.66E-04	1/14804	16	3.89E-04	1	6.62E-05	0	0	0	0	9	1.39E-04	0	0	1	9.41E-05	42	4.61E-02	0	0	0	0
	nonsense	5	67	4.53E-04	1/14804	31	7.53E-04	4	2.65E-04	0	0	4	8.32E-04	14	2.16E-04	1	3.03E-04	8	7.52E-04	1	1.10E-03	1	3.29E-03	3	1.48E-03
KMT2D	**Pathogenic**	**4**	**4**	**2.70E-05**	**1/18508**	**1**	**2.43E-05**	**0**	**0**	**1**	**2.01E-04**	**0**	**0**	**2**	**3.09E-05**	**0**	**0**	**0**	**0**	**0**	**0**	**0**	**0**	**0**	**0**
	frameshift	3	3	2.03E-05	1/24674	0	0	0	0	1	2.01E-04	0	0	2	3.09E-05	0	0	0	0	0	0	0	0	0	0
	nonsense	1	1	6.75E-06	1/74023	1	2.43E-05	0	0	0	0	0	0	0	0	0	0	0	0	0	0	0	0	0	0
	**Likely Pathogenic**	**28**	**37**	**2.50E-04**	**1/2644**	**11**	**2.67E-04**	**5**	**3.31E-04**	**3**	**6.03E-04**	**8**	**1.66E-03**	**9**	**1.39E-04**	**0**	**0**	**0**	**0**	**1**	**1.10E-03**	**0**	**0**	**0**	**0**
	frameshift	18	23	1.55E-04	**1/4113**	8	1.94E-04	3	1.99E-04	1	2.01E-04	5	1.04E-03	5	7.71E-05	0	0	0	0	1	1.10E-03	0	0	0	0
	missense	3	5	3.38E-05	**1/24674**	1	2.43E-05	1	6.62E-05	0	0	0	0	3	4.63E-05	0	0	0	0	0	0	0	0	0	0
	nonsense	4	5	3.38E-05	**1/18508**	1	2.43E-05	1	6.62E-05	2	4.02E-04	0	0	1	1.54E-05	0	0	0	0	0	0	0	0	0	0
	splice acceptor/donor	3	4	2.70E-05	**1/24674**	1	2.43E-05	0	0	0	0	3	6.24E-04	0	0	0	0	0	0	0	0	0	0	0	0
